# The three-spined stickleback as a model for behavioural neuroscience

**DOI:** 10.1371/journal.pone.0213320

**Published:** 2019-03-26

**Authors:** William H. J. Norton, Héctor Carreño Gutiérrez

**Affiliations:** Department of Neuroscience, Psychology and Behaviour, College of Life Sciences, University of Leicester, Leicester, United Kingdom; University Zürich, SWITZERLAND

## Abstract

The three-spined stickleback (*Gasterosteus aculeatus*) is a small teleost fish that is ubiquitous across the Northern Hemisphere. Among the behaviours that have been characterised in this species is ritualized courtship, aggressiveness and parental behaviour. Whereas three-spined sticklebacks have been used for ecological, evolutionary, parasitological and toxicological research, its complex behavioural repertoire and experimental advantages have not been exploited for basic neuroscience research. The aim of the present study is to describe some innate behaviours of laboratory bred three-spined sticklebacks by using a battery of tests that have been developed and validated to model some aspects of human psychiatric disorders in zebrafish. We recorded mirror induced aggression, novel object boldness, shoaling, and anxiety-like behaviour using both the novel tank diving and the black-white preference tests. We show that behaviour of three-spined sticklebacks in these standard tests is remarkably similar to that of zebrafish and other species and can be altered by fluoxetine and buspirone. These findings highlight the potential of using three-spined sticklebacks for cross-species and translational studies.

## Introduction

The three-spined stickleback (*Gasterosteus aculeatus*) is a small teleost fish that is ubiquitous across the Northern Hemisphere. Originally from marine environments, they colonised diverse fresh water systems thousands of years ago, which has made them ideal species to study evolutionary biology, adaptation and ecology [1]. Three-spined stickleback are easy to find in rivers, lakes and ponds as well as coastal regions. They are also easy to breed and maintain in the lab at low cost and the genome sequence is available (www.ensembl.org). The ethology of this species was extensively studied during the 20^th^ century thanks to the pioneering work of Nico Tinbergen [2,3]. Three-spined stickleback have also been used to study behavioural syndromes such as aggression-boldness and their implication for evolution [4–6]. Among the behaviours better characterised in this species are ritualized courtship, aggressiveness and parental behaviour. Sexually mature males develop a bright red throat and fore-belly called nuptial coloration. During the breeding season territorial males become aggressive and dominant. A male carefully builds a nest which he actively protects from conspecifics and also other species. When two males encounter they display several agonistic behaviours, including circling with the spines erected, charges and chases, bites to the opponents’ body and the eventual flee of the subordinate [7]. If a ripe female with a prominent silver belly approaches the male’s territory courtship proceeds following a chain of events. After a zig-zagging dance, the male leads the female to the nest and he shows the position of the entrance. Once the female is inside the nest the male stimulates spawning by quivering the female’s tail. After spawning the female leaves the nest and the male quickly fertilises the eggs [8]. When several clutches have been collected the parental phase starts. The male stays close to the nest to look after the offspring, fanning the eggs and protecting the newly hatched fish for several days [3].

One of the experimental advantages of these animals over other fish species such as zebrafish is the ease of tagging individuals and measuring consistent individual differences in behaviour over time and across different contexts [9]. In addition to inter-individual variation within a population, differences in behaviour have been reported between populations from different habitats. Differences in predation pressure in different environments affect physiological and behavioural responses [10]. Marine and benthic populations differ in schooling behaviour [11], and fish from streams and ponds show differences in boldness [12]. Moreover, the presence of water pollutants such as endocrine disruptors has an impact on behaviour [13]. Parasitism is another well-described source of variation in behavioural responses. Three-spined sticklebacks are the intermediate host of the tape-worm *Schistocephalus solidus*. Infected fish become bolder and therefore more likely to be eaten be predators allowing the parasite to complete its life cycle [14,15]. Uninfected fish also differ in their responses to predators, either real or simulated, with some individuals taking more risks than others [5].

Some of these behavioural studies have used lab bred animals, whereas the majority of them used fish caught from natural populations in harbours or freshwater systems. These fish are transferred to the lab where they are kept under controlled conditions that vary, with temperature and day-night cycle simulating natural conditions or adjusted as the study requires. Housing conditions also differ as fish are kept in different types of tanks with different kinds of enrichment. Some studies have been carried out using groups of fish, and others on single previously-isolated individuals. In some experiments, sexually mature adult fish have been tested (usually males) whereas in other cases sub-adults or fry have been used. The different methods and tests used by different labs have been described in great detail in scientific publications. However, there is a need for standardisation of procedures and behavioural tests in order to compare results across labs and design future studies based upon previous findings. Whereas three-spined sticklebacks have successfully been used for ecological, evolutionary, parasitological and toxicological research, its complex behavioural repertoire and experimental advantages have not been exploited in basic neuroscience research. This species has the potential to be used alongside other popular models to gain insight into the mechanisms underlying some diseases affecting behaviour. Studying the behaviour of the three-spined stickleback in standard tests that are relevant for human diseases, with good face and predictive validity, would be a starting point for cross-species and translational studies.

The aim of the present study is to describe some innate behaviours of laboratory bred three-spined sticklebacks by using a battery of tests that have been developed to model some aspects of human psychiatric disorders in zebrafish [16–18]. We have performed assays to measure mirror-induced aggression, novel object boldness, shoaling and anxiety-like behaviour, taking advantage of automated video tracking to generate the data. We discuss the similarities and differences in behaviour of the animals tested in our study with that reported by previous studies using similar paradigms. We also compare behaviour of sticklebacks and zebrafish highlighting some striking similarities.

## Materials and methods

### Animals and husbandry

In the present study we used three-spined sticklebacks (*Gasterosteus aculeatus*) that were the F3 of a freshwater population captured in the river Soar (Leicester, Leicestershire, UK) in 2014. The fish used in this study were randomly taken from a large population obtained by *in vitro* fertilisation in July 2017. Forty fish were housed in a 27 L tank on a circulating system (Xenoplus systems, Techniplast) in a dedicated fish facility at the University of Leicester. Flow rate was set at 2 tank changes an hour. Fish were reared under temperature and light-dark conditions simulating natural seasonal variation until March 2018 (S1 Table). From this point fish were kept in non-breeding conditions at 12 ± 1°C on a 12:12 h light:dark cycle (i.e. March conditions) for the duration of the experiments, that were conducted in June 2018. This permitted us to measure behaviour in non-reproductive individuals to avoid the confounding factors associated with territorial and courtship behaviours. As the fish we used had not reached sexual maturity males and females were visually identical. One week before the behavioural recordings fish were randomly caught and split in three 13.4 L tanks: two tanks containing 15 fish each and one tank containing 10 fish. These tanks were located in the central shelf of a large rack, so that all three received similar illumination and were surrounded by other tanks on the sides. No enrichment was provided. Fish were fed daily ad libitum with defrosted Chironomid larvae each afternoon at the end of the experiments. Fish were kept in accordance with institute guidelines for animal welfare. All work was conducted under a UK Home Office licence and was approved by a local Animal Welfare and Ethical Review Body (AWERB) committee at the University of Leicester.

### Behavioural methods

The behavioural experiments were conducted inside the fish facility to avoid the potential stress of transferring the animals into a different room. Recordings were conducted between 10:00 and 16:00 to minimise possible diel influences. The 13.4 L housing tank containing the 15 fish designated as the control group was taken out of the system and placed onto a trolley for 30 min before the experiments began. Single fish were netted and placed into the testing tank. After the experiment fish were collected in an intermediate tank and then placed back into the same 13.4 L housing tank and retested for a different behaviour 2 days later. Drug treatments were applied to fish located in the other two 13.4 L housing tanks. The one with 15 fish was used for buspirone treatment and the one with 10 fish for fluoxetine treatment. Fluoxetine hydrochloride (Cat. No. F4780, LKT Laboratories) was applied by immersion at a concentration of 5 mg/L for 3 hours before measuring behaviour [19,20]. Buspirone hydrochloride (Cat. No. 0962/100, Tocris) was applied by immersion at a concentration of 25 μM for 30 min before measuring behaviour [21]. The fish treated with either fluoxetine or buspirone were not reused for other tests to avoid any potential alteration of the brain neurochemistry that could affect behaviour. The number of sticklebacks tested (n = 10–15) was calculated using power analysis based upon pilot experiments that we carried out in three-spined sticklebacks and published studies in zebrafish [20,22]. Recordings were performed using FlyCapture2 2.5.2.3 software and two digital cameras from Point Grey Research. Ethovision XT8 video tracking software (Noldus) and were used to analyse most of the behavioural endpoints. The latency to the first attack, number of attacks, time spent biting, time spent freezing at the bottom of a novel tank, time spent in the white compartment and number of crosses in a black-white preference test were manually scored after replaying the videos in Windows Media Player (Microsoft). To avoid any bias, the videos were renamed and manually scored in a blind manner. We conducted the following experiments using tests and paradigms that have been established for zebrafish research.

### Mirror-induced aggression

Adult stickleback aggression was measured using mirror-induced stimulation as described for zebrafish in [20]. Single fish were placed in the middle of a glass tank (30 × 10 × 15 cm, L × W × H) filled with 10 cm water depth and immediately recorded from above for 5 min. Three walls of this setup were covered in a white opaque material. A mirror offset at an angle of 22.5° was placed outside the transparent wall to elicit aggression. One of the control fish got trapped in the net when it was being transferred into the testing tank and so we discarded it, meaning that we used 14 control fish and 10 fluoxetine treated fish. The time biting or pushing against the mirror and thrashing the tail fin was manually scored as time spent biting. We also manually quantified the latency to the first bite and the number of aggressive attacks as the number of times the fish approached the mirror from a distance larger than one body length to bite it. The total distance swum was quantified with Ethovision. Ten sub-adult fish of 3 months of age were simultaneously tested in the setup described in [23]. Single fish were placed in small tanks (9 × 4 × 4 cm, L × W × H) arranged in groups of four with three walls opaque and the fourth transparent to allow the interaction with the mirrors. Locomotion was also measured in this setup by covering the mirrors with an opaque divider. Fish were recorded from the top for 5 min and the analysis was automatically obtained with an extension of ZebraLab software (VpCore2) developed by ViewPoint Behavior Technology. Aggression and locomotion are expressed as aggression units and locomotion units. These are arbitrary units that correlate well with time spent in aggressive display (i.e. directing bites to the mirror image and thrashing the tail, quantified manually) and with total distance swum (quantified using Ethovision) as described for juvenile zebrafish in [23]. We also measured aggression manually and calculated its correlation with the automatic score.

### Novel object boldness

Novel-object boldness was measured using the setup described in [20]. The testing tank (40 × 25 × 15 cm, L × W × H) had a 15 ml Falcon tube filled with dark blue and yellow modelling clay suspended midway in the water column at one end used as the object. This setup was filled with 10 cm of water and the walls were surrounded by white opaque material to minimise reflection. Single fish were placed into the setup and recorded from the top for 5 min. We used Ethovision to quantify the time spent within one body length of the novel object, the latency to the first approach, and the total distance swum. We used 15 fish.

### Shoaling

Shoaling was measured in 6 groups of 5 individuals swimming in a large opaque plastic tank (80 × 60 × 60 cm, L × W × H) filled with water to a depth of 10 cm. A group was placed in the tank a left to explore the tank for 20 min. After this period of habituation the fish were recorded from the top for 10 min. The nearest neighbour and inter-individual distances were measured using VpCore2 (ViewPoint Life Sciences).

### Novel tank diving test (NTT)

We performed the NTT to measure anxiety-like behaviour in a standard 3.5 L trapezoid tank (27.9 × 22.5 × 11.5 × 15 cm, L (top) × L (bottom) × W × H) similar to the 1.5 L trapezoid widely used for zebrafish [22,24–26]. The only difference was that our tank was slightly wider accounting for the larger size of sticklebacks. Single fish were placed in this setup and recorded from the side for 5 minutes. The tank was divided in three zones—bottom, middle and top–for the analysis of the videos with Ethovision. We automatically measured the amount of time spent on the top third of the tank and total distance swum. We manually quantified freezing as cessation of swimming while at the bottom of the tank, as three-spined sticklebacks can hover in the middle of the water column. We used 15 fish as controls and 15 fish treated with buspirone.

### Black-white preference test

This setup consisted of a transparent plastic tank (30 × 20 × 12 cm, L × W × H) with the bottom and walls surrounded by two removable opaque plastic covers that divided the tank into two zones: black and white. This set is also used for zebrafish behavioural studies [27,28]. The water depth was 10 cm. Single fish were transferred into the white half and recorded from above for 5 min. We manually quantified the total time spent in the white zone and the number of crosses from the black to the white zone. We considered a cross when the pectoral fins were in the white half of the tank. We used 15 fish as controls and 15 fish treated with buspirone.

### Data analysis

Data from Ethovision and manual analysis were collected in Excel (Microsoft) and statistical analyses were carried out with GraphPad Prism7. The graphs show the mean and the standard error of the mean. Each dot represents one fish. Data were assessed for normality using D’Agostino & Pearson normality test. Homocedasticity was tested using Levene’s test. We used unpaired Student’s t-tests or Mann Whitney U tests as appropriate for comparison of drug treated fish and controls. We also used the Pearson’s correlation coefficient. The significance level was set at 5%.

## Results

### Mirror-induced aggression

**Fig 1:**
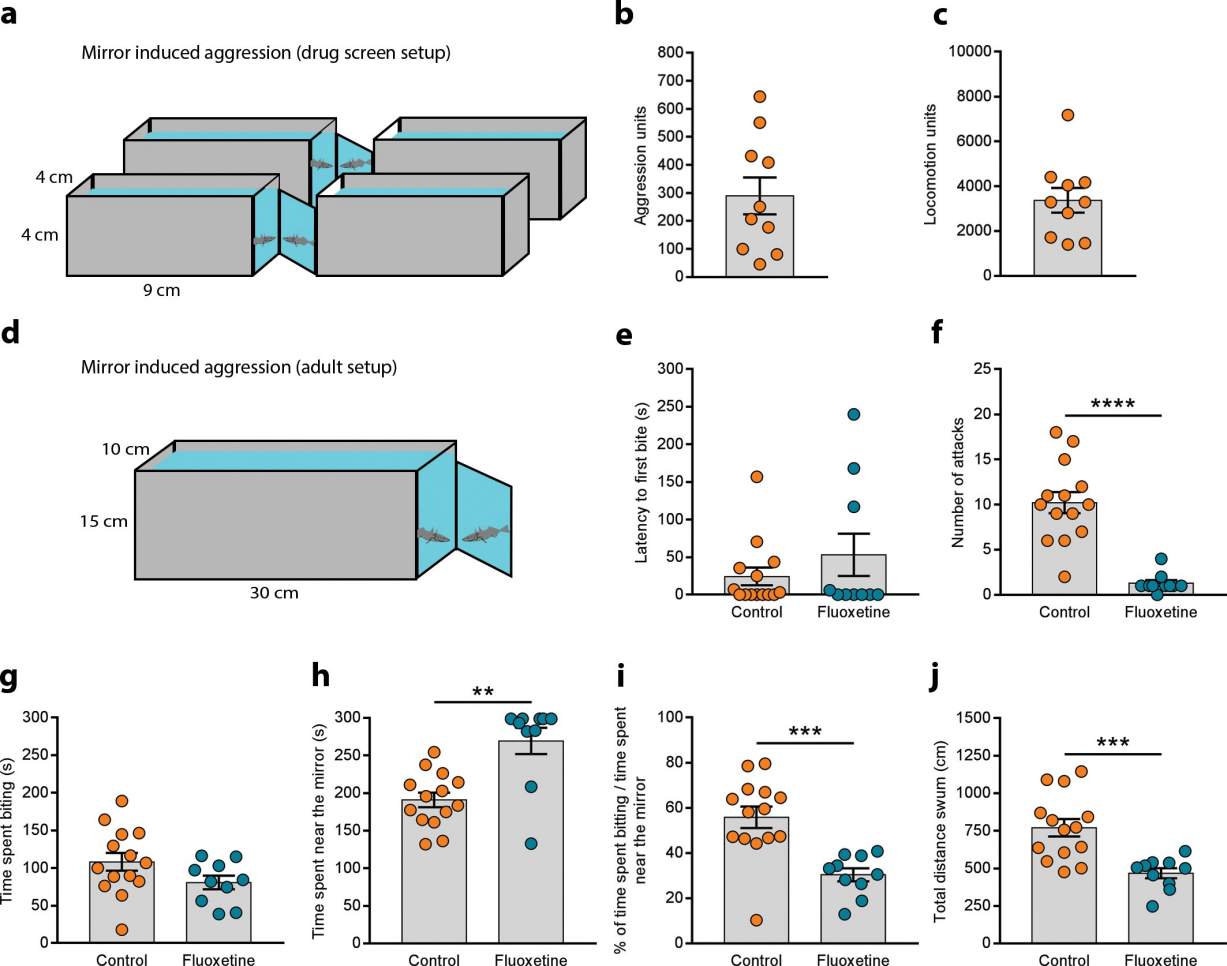
Aggression test. **(a)** Set-up to measure mirror induced aggression in sub-adult fish. **(b)** Aggression units and **(c)** locomotion units measured in 3 month old fish. **(d)** Mirror induced aggression test for adult fish. **(e)** Latency to first bite, **(f)** total time spent biting, **(g)** number of attacks, **(h)** time spent near the mirror, **(i)** time spent biting when swimming near the mirror and **(j)** total distance swum by control and fluoxetine treated fish in this set-up. ** p < 0.01, *** p < 0.001, **** p < 0.0001.

We tested aggression in 10 sub-adult fish of 3 months of age in the setup described in [23] where multiple individual can be recorded simultaneously in small arenas increasing the throughput of the experiment (Fig 1A). This setup is connected to software that automatically quantifies aggression. All the fish interacted with their own mirror image and displayed aggressive postures. The average aggression levels were 289.6 ± 65.66 aggression units (Fig 1B). The manual quantification of these videos revealed a good correlation with the values obtained by the software (Pearson’s r: 0.8259, p = 0.0032), yielding 100.71 ± 21.33 s, which is very similar to the values obtained in adults (Fig 1G). The software also measures the distance swum by the fish in the testing tanks before the mirror is presented as a readout of general locomotor activity (Fig 1C). This protocol to measure aggression automatically in young fish can be used to screen for different drugs that modify this behaviour [23].

We used the mirror-induced aggression test to quantify aggressive behaviour of adult fish in the mirror induced aggression test as described in [20] (Fig 1D). When single fish were placed in the testing tank some on them immediately approached the mirror and started biting and chasing their own reflection (Fig 1E). Although a few fish had a higher latency to approach, all of the 14 fish tested responded to their own mirror image attacking as if there was an opponent present. The repeated biting was accompanied by pushes towards the mirror and thrashes of the caudal fin resulting in undulating body movements. The total duration of this aggressive display was 108.2 ± 11.85 s (mean ± SEM) (Fig 1F), split into several attacks alternating with exploration of the rest of the tank. From the middle and back of the tank fish directed an average of 10.21 ± 1.17 short fast attacks to the mirror that ended with a bite towards its own reflection (Fig 1G). Aggression can be modulated by drugs that interact with 5-HT neurotransmitter signalling in zebrafish [29]. The selective 5-HT reuptake inhibitor fluoxetine decreases aggression in this species [19,20]. We treated three-spined sticklebacks with this drug and recorded their behaviour in the mirror induced aggression test hypothesizing that it would decrease aggression. Fluoxetine did not alter the latency to the first bite (Mann Whitney test: U = 70, p > 0.9999; Fig 1E) or the total time spent biting the mirror and thrashing the tail (Student’s t-test: t_(22)_ = 1.717, p = 0.1000; Fig 1F). However, the number of attacks was reduced in comparison with the control untreated fish (Mann Whitney test: U = 1.5, p < 0.0001; Fig 1G). Interestingly, fluoxetine treated fish spent more time near the mirror (i.e. within on body length) than control fish (Mann Whitney test: U = 18, p = 0.0014; Fig 1H). However, the percentage of time spent displaying overt aggression when swimming near the mirror was reduced in the fluoxetine treated group (Student’s t-test: t_(22)_ = 4.178, p = 0.0004; Fig 1I) suggesting a decrease in aggression. The total distance swum was also smaller upon fluoxetine treatment (Student’s t-test: t_(22)_ = 4.027, p = 0.0006; Fig 1J).

### Novel object boldness

**Fig 2:**
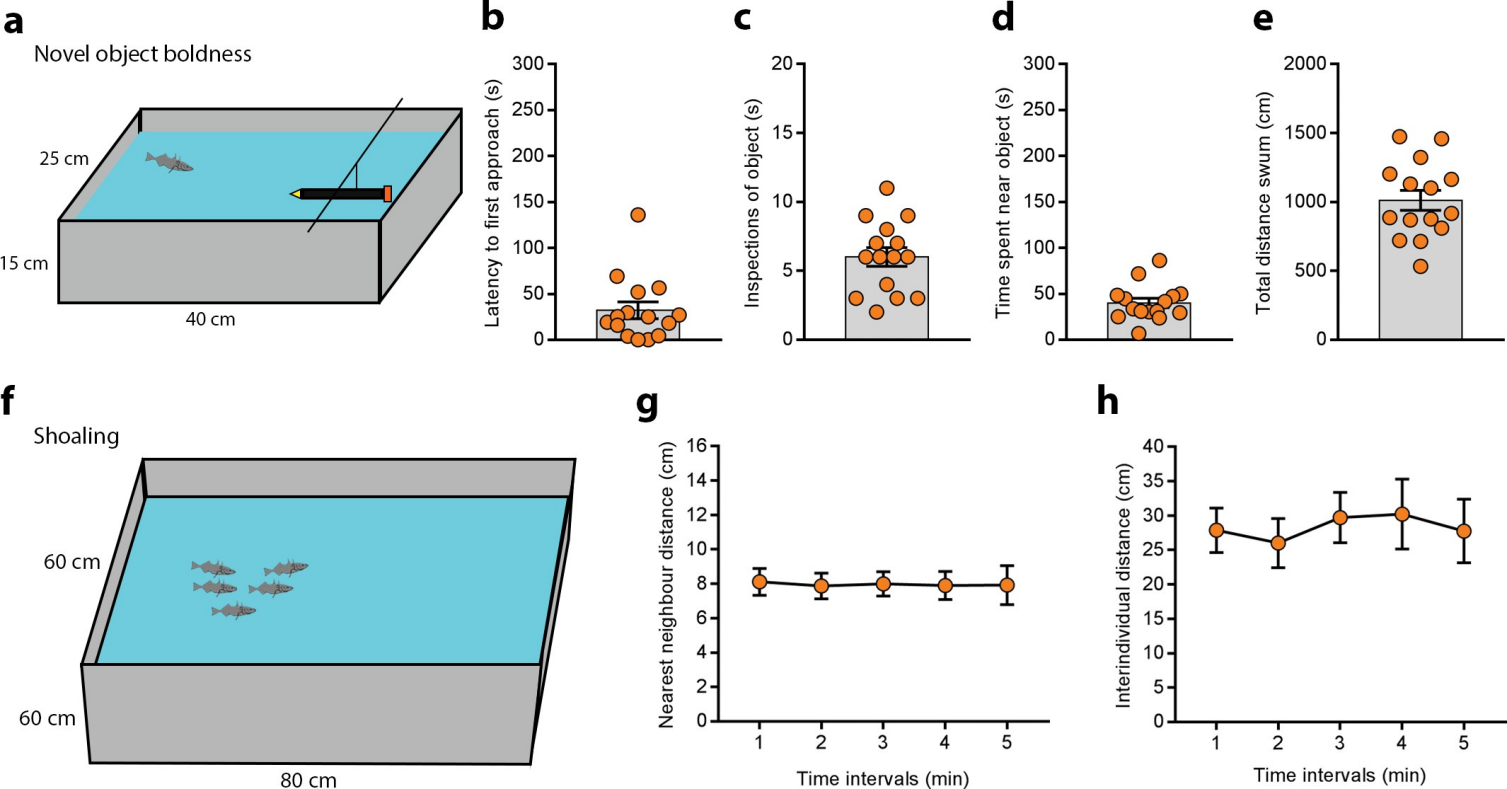
Boldness and shoaling tests. **(a)** Novel object boldness test. **(b)** Latency to first approach to the novel object **(b),** number of inspections **(c)**, total time spent close to the object **(d)** and total distance swum **(e)** by adult fish tested in this setup. **(f)** Shoaling test. **(g,h)** Nearest neighbour- and inter-individual distance measured in this tank. Each dot on the scatter plots represents one fish.

We tested 15 fish in the setup described in [20] where a 15 ml centrifuge tube filled with clay is suspended in the water at one side of a tank (Fig 2A). The latency to first approach the novel object was 32.17 ± 9.143 s (Fig 2B) and was followed by an average of 6 ± 0.676 approaches (Fig 2C). The total time spent within 1 body length of the object was 39.99 ± 5.071 s in a 5 min experiment (Fig 2D). The time spent near the novel object could be a result of random exploratory activity across the whole tank, with more active fish swimming more time close to the object, without a specific motivation (i.e. novel object approach or avoidance). We measured the total distance swum in the testing tank (1012 ± 72.35 cm (Fig 2E)) and calculated the correlation between general locomotor activity and time spent close to the object. Interestingly, there was no correlation between these two parameters (Pearson’s r = 0.1183, p = 0.6745). There was also no correlation between the time spent near the object and the time spent in an area opposite the object (Pearson’s r = -0.0244, p = 0.9312).

### Shoaling

Several studies have quantified the tendency of a focal three-spined stickleback to approach and stay close to a group of conspecifics placed behind a transparent barrier in different contexts [30–32]. However, to our knowledge there are no studies measuring shoaling in free swimming sticklebacks by calculating the distances between the members of the group. Nearest neighbour (NND) and inter-individual (IID) distances are two measures of shoal cohesion. We tested 6 shoals of 5 fish each placing them in a large tank (Fig 2F). After a 20 min habituation period where the fish explored the tank we recorded from the top for 5 min. We used ViewPoint software to automatically track the individuals and determine the distances between them. The average NND was 7.965 ± 0.656 cm and remained stable over time (Fig 2G). The average IID was 28.32 ± 3.383 cm and was also quite stable (Fig 2H).

### Novel tank diving test

**Fig 3:**
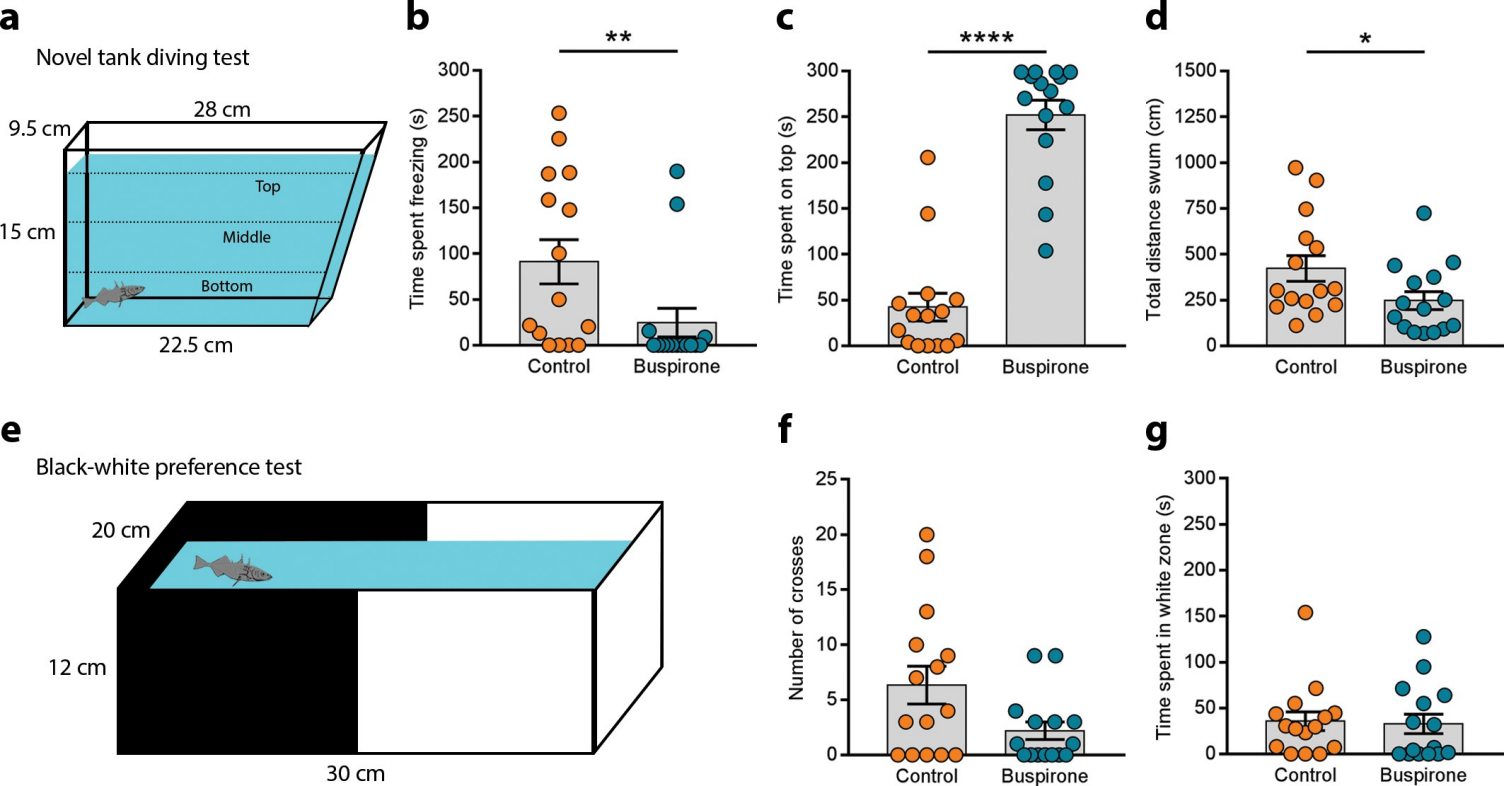
Novel tank diving and black white preference tests. **(a)** Novel tank diving setup. **(b)** Time spent on the top third of the tank, **(c)** freezing at the bottom and **(d)** total distance swum in control and buspirone treated fish. **(e)** Black white preference test. **(f)** Number of crosses from black to white and **(g)** total time spent in the white zone of control and buspirone treated fish in this set-up. Each dot on the scatter plots represents one fish. * p < 0.05, ** p < 0.01, **** p < 0.0001.

We used the novel tank diving test to measure anxiety-like behaviour (Fig 3A). This set-up has been extensively validated in zebrafish by applying drugs that are known anxiolytic or anxiogenic. The first drug used was the 5-HT receptor 1A partial agonist buspirone, a well-known anxiolytic compound which strongly reduces anxiety-like behaviour in zebrafish in a dose dependent manner [21,33]. In a non-treated group of fish the NTT elicited a strong geotaxic response, where fish immediately dived to the bottom third of the tank. Eleven out of 15 fish showed freezing behaviour, which is a cessation of movement except for the opercula and the mouth. The average duration of these freezing episodes was 91.04 ± 24.07 s (Fig 3B). When fish were freezing they also erected their dorsal fins and bent their tails upwards in a J-shape. After the initial bout of freezing, fish gradually explored of the top third of the tank. The total time spent swimming on the top was 42.24 ± 15.15 s in a 5 min experiment (Fig 3C). Freezing and geotaxis are indicators of stress responses and anxiety in a potentially threatening context. Using video-tracking we can also automatically quantify the total distance swum (Fig 3D) as a readout of exploratory activity. To test the face validity of this test in three-spined sticklebacks–i.e. its ability to measure anxiety—we applied 25 μM buspirone by immersion for 30 min. We hypothesised that this anxiolytic drug would cause a decrease in the anxiety-like behaviour measured in this test. Only 4 out of 15 fish displayed freezing upon buspirone treatment, a significant decrease compared to untreated fish (Mann-Whitney test: U = 54, p = 0.0089; Fig 3B). Buspirone-treated fish spent 251.9 ± 16.13 swimming at the top of the tank, which is a dramatic increase compared to the untreated fish (Mann-Whitney test: U = 5, p < 0.0001; Fig 3C). The strong decrease in freezing and geotaxis indicate a sharp reduction of anxiety-like behaviour. Total distance swum was slightly reduced (Mann-Whitney test: U = 64, p = 0.0367; Fig 3D).

### Black-white preference test

We further characterised anxiety-like behaviour using the black-white preference test (Fig 3E). This test has been used in some fish species, including zebrafish, to measure the preference for a black zone when an individual experiences the conflict of choosing between black or white [27]. It has also been validated with a number of drugs such as buspirone. We placed individual fish in the white zone and measured the latency to enter the black zone, the number of transitions from black to white and the time spent in the white zone. The number of transitions was on average 6.33 ± 1.71 (Fig 3F). Most of these transitions were short bouts of rapid swimming into the white side followed by a turn back into the black side. However, some fish spent long periods of time in the white zone immobile. The total time spent in the white zone was 35.65 ± 10.12 s (Fig 3G) indicating a strong preference for staying in the black side or scototaxis, suggesting anxiety-like behaviour. We treated the fish with 25 μM buspirone in system water for 30 min reasoning that this could increase the number of transitions into the white side or the amount of time spent in this area. However, there were no differences between treated and untreated fish in the number of transitions (Mann-Whitney test: U = 74; p = 0.1006; Fig 3F) or the time spent in the white zone (Mann-Whitney test: U = 101, p = 0.6420; Fig 3G).

## Discussion

In this paper we have described the behaviour of laboratory bred three-spined sticklebacks using paradigms that are relevant to model some aspects of human psychiatric disorders in other species. We have described aggressive behaviour using the mirror-induced aggression set-up. We have also tested novel object boldness and measured group cohesion within a shoal. Finally, we measured anxiety-like behaviour using both the novel tank diving and the black-white preference tests. All these tests have been validated for zebrafish, a popular model for behavioural neuroscience, and can be used to measure behaviour across fish species.

Aggressive behaviour in territorial males has been documented decades ago. When a male establishes its territory during the breeding season it defends its boundaries to keep intruders away by chasing and biting them [7,34]. A similar aggressive response can be experimentally elicited by placing an intruder confined in a transparent container into a tank occupied by a territorial male [3,35]. An alternative approach to test fish aggressiveness is quantifying the responses of an experimental fish to its own mirror image.

Mirror stimulation has been extensively used during the last decades in many species such as the Siamese fighting fish [36], *Astatotilapia burtoni* [37], coho salmon [38], mangrove killifish [39], rainbow trout and guppies [40], Mozambique tilapia [41] and zebrafish [20,29,42]. Despite the biological differences between evolutionary and ecologically distant fishes, they all display aggressive postures and direct bites to their mirror images indicating that aggression is a highly conserved behaviour among teleost fishes. However, the differences in set-ups, scoring methods and analysis reported in different studies makes it challenging to compare data between species.

Some recent studies have already described mirror induced aggression in male three-spined sticklebacks. A study by [43] showed that isolated territorial males that were actively building their nests approached and attacked a mirror placed in their tanks. When individual fish were placed in large tanks with three mirrors covering the walls they also displayed aggression, although some fish did not respond to this stimuli [44]. Kellner and colleagues have also used the approach of lowering a mirror into a tank to measure attack behaviour in adult females [31]. In a later study they tested adults of both sexes prior to sexual maturation by lowering a mirror into a tank occupied by single fish, although some fish did not discover the mirror and were omitted [45].

We demonstrate here that the set-up routinely used in zebrafish research, with three opaque walls and an inclined mirror outside the tank, is a suitable and reliable method to measure three-spined stickleback aggression. The opaque walls in our setup ensure that all fish see the mirror stimulus without the need of exploring a large area and the presence of other confounding stimuli in the tank. Moreover, we have observed in our lab that when a mirror is presented outside of a plastic transparent tank fish respond less to this stimulus and bite the walls instead (unpublished observations). In our experiment all the individuals–sub-adults and adults- responded to the mirror stimulus by approaching and displaying attack behaviour.

The feasibility of testing sub-adult fish in this setup coupled to automatic quantification [23] can be used to perform screens to test the effect of drugs or mutations on aggression in both zebrafish and three-spined sticklebacks. When manually scoring adult fish we quantified the total amount of time the fish spent in active aggressive display, biting and thrashing their tail (i.e. time spent biting) [42]. We favour this method instead of counting individual bites or attacks as previous studies have done as bites are difficult to quantify and attacks do not represent the duration of the aggressive interaction. Zebrafish are more active and so they attack the mirror more intensively, whereas three-spined sticklebacks swim slower, which affects the intensity of the aggressive interaction, although we cannot quantify this at present. However, the total time that our sticklebacks spent in aggressive display was slightly higher than those reported in zebrafish studies [20,22,23].

When applied acutely, the specific 5-HT reuptake inhibitor fluoxetine reduces zebrafish aggression both in the mirror test [20] and in a real opponent paradigm [19]. Acute treatment with fluoxetine also reduces aggression directed against dummy fish [19] or to the mirror image [46] in male Siamese fighting fish. Both acute and chronic treatment with fluoxetine also decreases aggression in the bluehead wrasse [47]. Thus, the decrease in aggression upon fluoxetine treatment that we report in the present study is in line with previous findings in other fishes and supports the role of this drug in controlling aggression. Other substances such as deprenyl [22], ethanol [42] or lithium carbonate [48] also decrease aggression in zebrafish and can be tested in three-spined sticklebacks. Understanding the genetic and pharmacological basis of this behaviour in different species may shed light on conserved mechanisms underpinning pathological levels of aggression.

Boldness is the propensity to engage in risk taking behaviour as opposed to shyness. Aggression and boldness are key components of the aggression-boldness behavioural syndrome, which was described in three-spined sticklebacks some decades ago [4]. Boldness has been extensively characterised in sticklebacks using different paradigms. One approach is to test a fish’s responses to the presence or the attack of a predator–either real or simulated [5]. Using this method, some studies have measured the latency to resume eating after an attack [5,9,12], the amount of time freezing after an attack [49] or the number of inspections and orienting to a predator [50]. The tendency to leave a shoal is also an indicator of boldness [9].

An alternative and easy to perform approach is to measure the response of a fish to a novel object present in the tank. The behavioural endpoints used to score this type of behaviour include recording the position of the fish 2.5 times per second for 10 minutes to obtain the mean distance to a little statue [51]; manually recording the time spent freezing when a glass flask is introduced into the tank [52]; measuring the distance to a plastic toy every 20 seconds during 10 minutes [53]; or quantifying the time spent in 3 concentric circles surrounding a plastic toy pig [31]. These methodologies are all based on manual quantification which implies lengthy analysis and less accurate measurements than those obtained by video tracking. Moreover, fish used in different studies were caught from different habitats (i.e. a freshwater pond, a creek or a harbour) which adds potentially confounding factors since predator pressure in different habitats may impact behavioural responses [10]. The different methodologies and behavioural endpoints recorded, and the different populations studied, hamper the comparison of results between different labs.

In this paper we have tested boldness as the amount of time spent near a novel object as previously described in zebrafish studies [20,54]. Quantifying the actual time the fish spends near the object by automated video tracking gives an accurate endpoint that is easy to compare between laboratories. Furthermore, recording the total distance swum in this set up gives a readout of the general exploratory activity of the fish in this test. The non-existent correlation between the time spent near the object and the time spent in an area of equal size opposite to the object suggests that the behaviour of the fish is driven by the object; bold fish spend more time close to the object than in the empty area at the other end of the tank. In line with this, the absence of a correlation between time spent near the object and total distance swum suggests that exploration of the novel object is not simply due to higher locomotor activity in the whole tank. The novel object boldness test can complement other tests used to characterise this behaviour.

Shoaling is the tendency of fish to form groups of conspecifics. This aggregation provides benefits for their members such as reduced predation risk and increased foraging efficiency and mating opportunities [55]. In three-spined sticklebacks this behaviour is typically measured in tanks with three compartments separated by transparent walls, where a focal fish in the centre shows preference to swim close to conspecifics located in one of the lateral compartments [30–32,56,57]. The same type of attraction is elicited if the conspecifics are confined in a glass flask placed in the tank with the focal fish [52,58]. When a shoal is moving synchronously and becomes polarized so that its members swim in the same direction it is named a school [59]. An ingenious paradigm has been implemented to manually quantify the tendency of a focal three-spined stickleback fish to follow a group of model conspecifics that swim in circles in a large tank forming a school [11,60]. However, these kind of tests do not actually measure the cohesion of the group members and do not reflect the natural characteristics of a free swimming shoal.

Nearest neighbour (NND) and inter-individual distance (IID) are two commonly used parameters to measure social cohesion in zebrafish [33,59,61–64]. NND is the average of the distance between any fish of the shoal and its closest neighbour. IID is the average of all the distances between the members of a shoal. Whereas IID precisely estimates the cohesion of a group as it considers all the distances it is affected by group size, and this must be accounted for if groups of different sizes are compared. IID can also be substantially modified by a single fish that leaves the shoal for some time. If a large group of fish splits into two or more subgroups during the behavioural recordings this will affect the average IID; however the NND will remain similar as long as there is cohesion in the subgroups. Therefore, these two measures are complementary but not inter-changeable.

Both the NND and IID can be calculated using software that allows automated tracking of multiple individuals. Miller and Gerlai developed the first kind of this software which opened up the possibility of measuring different parameters to assess changes in shoal density over time [63,65]. Since then, different software and refined approaches are being created to accurately measure the dynamics of a group. For example, Ethovision offers software to track multiple fish [66], and more sophisticated tools such as idtracker (www.idtracker.es/idsocial) can measure in great detail the interactions between fish swimming together whilst identifying each individual within a group [67,68]. In the present study we have used ViewPoint software (VpCore2) to measure NND and IID in groups of 5 fish swimming in a large tank. As expected, both parameters can be obtained in three-spined sticklebacks. We observed that sometimes one or two fish leave the group for a few seconds to explore the tank. In a group of 5 fish, when one individual leaves the group it alters 4 of the total 10 inter-individual distances contributing to 40% of the IID. If two individuals leave, they will account for 70% of the IID. This could explain the greater fluctuations in IID compared to the more stable NND.In zebrafish, shoaling can be impaired by some drugs such as nicotine or ethanol [69], phencyclidine (PCP), 3,4-methylenedioxymethamphetamine (MDMA), or reserpine [70], MK-801 [33], valproate [71], or scopolamine [66]. Both pharmacological manipulations and genetic studies can potentially be implemented in three-spined sticklebacks, making these species a promising tool for comparative and translational neuroscience.

Novel tank diving is a robust test used to measure anxiety-like behaviour in zebrafish [21,25,72]. In this paradigm fish prefer to stay at the bottom of a novel tank and avoid the top, which is perceived as threatening. A recent study measured bottom preference or geotaxis in adult female three-spined sticklebacks captured in a harbour [31]. The novel tank had three walls covered with black plastic and was divided into a bottom and top by a line. Behavioural parameters such as time spent in the top half, latency to first entry on top, time spent freezing and general locomotion were scored manually [31]. We suggest that there could be several issues with this setup. First, the large dimensions of the testing tank are likely to elicit horizontal exploratory swimming and thigmotaxis. A narrower tank would create a greater conflict between swimming on the potentially threatening top versus staying in the safer bottom. Second, the authors used a period of 20 min of habituation in a small container prior the recording in the novel tank. This confinement is potentially stressful and could affect the response of the fish by adding a stressor prior to testing. Moreover, we consider that the division of the tank into thirds instead of halves gives more accurate results, since some anxious or stressed individuals may cross the middle line and yet never swim on the top (close to the surface). Recording behaviour in three areas increases the power to detect subtle differences in anxiety between individuals. In line with this, video tracking of the fish would allow to better assess locomotor activity and speed up the analysis of all the behavioural endpoints.

We performed the novel tank diving test in a narrow tank without previous habituation and found a strong scape response. Fish immediately dived to the bottom where they swam and froze. Bottom preference and freezing often co-vary and are indicators of stress reactivity or anxiety-like behaviour. As the experiment progresses and fish become habituated to the tank they tend to gradually spend more time in the top third, a response typically observed in zebrafish [26]. Although the swimming pattern differ in these two species the preference for the bottom of the novel tank is remarkably similar. Three-spined sticklebacks tend to hover in the middle of the tank and move vertically. However, freezing should be only scored as lack of movement at the bottom of the tank (see Materials and Methods). Diving responses, bottom dwelling and freezing have also been observed in other species such as guppies [73,74] and Endler guppies [75].

Zebrafish studies have demonstrated that anxiety-like behaviour is controlled by 5-HT. Drugs targeting different components of the 5-HTergic pathway modulate behaviour in these two tests [28,76]. One of these drugs is the 5-HT receptor 1a partial agonist buspirone, which acts as anxiolytic in zebrafish decreasing geotaxis [21,76]. We have found that this drug strongly decreases geotaxis in three-spined sticklebacks, and reduces both the time spent freezing and the distance swam, which indicates a reduction of anxiety-like responses in agreement with zebrafish studies.

Scototaxis is an innate behaviour that has been observed in some fishes including zebrafish by using the black-white preference test [27]. Zebrafish strongly avoid the white compartment and stay in the black side most of the time, although some differences in this behavioural pattern have been reported in fishes from other taxa [77]. Scototaxis has been recently described in adult three-spined sticklebacks of mixed, unknown age and sex, caught in a freshwater pond [51]. In this study the authors used a grey open tank with two small white- and black-bottomed areas at one end and counted the number of times a single fish entered into each. Fish entered more often into the black area, demonstrating scototaxis in this species. However, there are two weaknesses in this set-up. First, fish can also choose not to enter any of these areas. Secondly, instantaneous recording of the number of counts every ten seconds does not accurately inform about the actual time spent in each area. The same study measured behaviour in a new combined diving and scototaxis test [51]. In this set-up there is a divider with a gap between the white and black half, adding an extra conflict. Thus, this test measures geotaxis, scototaxis, and also emergence and exploratory activity making the results difficult to interpret.

Whereas both the novel tank diving and the black-white preference tests are widely used to measure anxiety in zebrafish, they are complementary and measure different aspects of behaviour [78]. This could also be the case for three-spined sticklebacks and it is a possibility worth investigating in future studies. Here we have used a black-white test in which fish have to respond to the conflict between taking the risk of exploring the white half where they are more exposed or hide in the black area where they can camouflage. The strong scototaxis response we observed in our set-up could be explained by the dark colour of this species, especially in the dorsal part. Some individuals showed freezing behaviour, which is indicative of anxiety [79]. Thus, there is a striking similarity in the response of these two species in the black-white test. However it has been reported that scototaxis differs between species. For example, strong black preference is shown by guppies and cardinal-tetras, whereas no preference has been observed in the Nile tilapia or mosquitofish [27,77].

We applied buspirone expecting to see a decrease in scototaxis in line with the anxiolytic effect observed in the novel tank diving test. Moreover, buspirone has been shown to decrease scototaxis and geotaxis in zebrafish [76]. However, we did not observed any effect on scototaxis. It Is possible that a higher concentration of drug or a longer treatment is required to increase the time spent in the white zone. Alternatively, it may be that buspirone does not affect scototaxis in the same way as it affects diving responses in three-spined sticklebacks, because these behaviours are controlled by different 5-HT receptors or pathways.

Some serotonergic drugs do not have the same anxiolytic or anxiogenic effect on zebrafish in both tests. For example para-chlorophenylalanine (pCPA) is anxiogenic in the novel tank diving test and anxiolytic in the black-white preference test, and fluoxetine has the opposite effect in these two tests [76]. This emphasises the need to consider these tests as complementary due to their different sensitivity to the same pharmacological manipulations. The novel tank diving and the black-white preference tests elicit escape responses to perceived potential threats, such as predator attacks, in the form of bottom and black preference, in both zebrafish and three-spined sticklebacks. Interestingly, the same context (i.e. testing tank) elicits similar responses in both species. Further pharmacological validation of these tests is likely to make them powerful tools for biomedical research and drug discovery.

### Final remarks

Three-spined sticklebacks and zebrafish exhibit species-specific features and biological differences due to their evolutionary distance. The zebrafish is a tropical species that actively swims at regular speed by using the thrust of its caudal fin. Three-spined sticklebacks live in cooler habitats, are less active, and alternate bursts of swimming using its pectoral fins with episodes of immobility in the middle of the water column. However, both are amenable for behavioural testing in the lab and display very similar innate behaviours in the same tests. The availability of three-spined stickleback as a new fish model to perform comparative studies and translational research represents a valuable tool to further our knowledge about brain malfunction leading to neurobiological diseases.

The wealth of knowledge about stickleback behaviour obtained in the past decades, the consistent individual differences in behaviour and the availability of standard and easy to perform tests can be readily used to study human psychiatric disorders and search for novel treatments. Further validation of these tests using pharmacological approaches and measuring physiological responses, characterisation of complex behaviours such as learning and memory, attention, impulsivity or cognition, and neuroanatomical studies describing brain areas and circuits controlling behaviour will open up new avenues for cross species studies and translational research.

## Supporting information

S1 Dataset (XLSX)

S1 Table Light-dark and temperature conditions under which fish were reared until the behavioural recordings. (DOCX)
