# Correction: The three-spined stickleback as a model for behavioural neuroscience

**DOI:** 10.1371/journal.pone.0216518

**Published:** 2019-05-01

**Authors:** William H. J. Norton, Héctor Carreño Gutiérrez

The second author’s name appears incorrectly in the citation. The correct citation is: Norton WHJ, Carreño Gutiérrez H (2019) The three-spined stickleback as a model for behavioural neuroscience. PLoS ONE 14(3): e0213320. https://doi.org/10.1371/journal.pone.0213320.

## References

[1] Norton WHJ, Gutiérrez HC (2019) The three-spined stickleback as a model for behavioural neuroscience. PLoS ONE 14(3): e0213320 10.1371/journal.pone.0213320 30913214 PMC6435232

