# Supplementary material for: The three-spined stickleback as a model for behavioural neuroscience
Source: PLoS One. 2019 Mar 26;14(3):e0213320. doi: 10.1371/journal.pone.0213320 (PMC6435232; doi:10.1371/journal.pone.0213320)
Supplement: S1 Table — (DOCX) [file pone.0213320.s002.docx]

| **Date** | **Sunrise** | **Sunset** | **Day length** | **Temp °C** |
| --- | --- | --- | --- | --- |
| 01-jul | 4:45 | 21:31 | 16:45 | 18 |
| 14-jul | 4:58 | 21:22 | 16:24 | 19 |
| 01-Aug | 5:24 | 20:57 | 15:33 | 19 |
| 14-Aug | 5:45 | 20:32 | 14:47 | 18 |
| 01-Sep | 6:15 | 19:53 | 13:37 | 17 |
| 14-Sep | 6:37 | 19:22 | 12:45 | 16 |
| 01-Oct | 7:06 | 18:42 | 11:35 | 15 |
| 14-Oct | 7:28 | 18:12 | 10:43 | 14 |
| 01-Nov | 7:01 | 16:34 | 9:33 | 12 |
| 14-Nov | 7:25 | 16:12 | 8:47 | 10 |
| 01-Dec | 7:53 | 15:53 | 8:00 | 8 |
| 14-Dec | 8:08 | 15:49 | 7:40 | 6 |
| 01-Jan | 8:15 | 16:00 | 7:44 | 6 |
| 14-Jan | 8:08 | 16:18 | 8:09 | 8 |
| 01-Feb | 7:45 | 16:50 | 9:04 | 8 |
| 14-Feb | 7:22 | 17:15 | 9:52 | 10 |
| 01-Mar | 6:50 | 17:43 | 10:53 | 11 |
| 14-Mar | 6:20 | 18:06 | 11:46 | 12 |

**S1 Table. Light-dark and temperature conditions under which fish were reared until the behavioural recordings.** Experiments were conducted under the conditions set on 14^th^ March.
